# Response of patients with chest tightness variant asthma with routine asthma treatment regimen: A 1‐year multicenter, prospective, real‐world study

**DOI:** 10.1002/ctm2.178

**Published:** 2020-09-15

**Authors:** Fugui Yan, Wen Li, Wei‐jie Guan, Min Chen, Chen Qiu, Wei Tang, Xiansheng Liu, Xudong Xiang, Jing Li, Meiling Jin, Yuanrong Dai, Ping Chen, Xiaohong Wu, Zhongmin Qiu, Liang Dong, Limin Zhao, Xiaoping Lin, Changgui Wu, Bin Wu, Yalian Yuan, Fei Shi, Ting Zhang, Jun Zhou, Min Xie, Xiaoyu Fang, Hongliang Zhang, Bing Xiao, Mo Xian, Jian Wang, Zhangwei Qiu, Jie Lin, Bingbing Ji, Yong Zhou, Yu Li, Chunhong Liu, Yiping Chen, Yiming Zeng, Lingli Liu, Wen Hua, Huaqiong Huang, Jiesen Zhou, Yue Hu, Luanqing Che, Songmin Ying, Zhihua Chen, Nanshan Zhong, Huahao Shen

**Affiliations:** ^1^ Key Laboratory of Respiratory Disease of Zhejiang Province Department of Respiratory Medicine Second Affiliated Hospital of Zhejiang University School of Medicine Hangzhou China; ^2^ Department of Respiratory Medicine State Key Laboratory of Respiratory Disease Guangzhou Institute for Respiratory Health First Affiliated Hospital of Guangzhou Medical University Guangzhou China; ^3^ Department of Respiratory Medicine Affiliated Hospital Guangdong Medical University Zhanjiang China; ^4^ Department of Respiratory Medicine Shenzhen People's Hospital Shenzhen China; ^5^ Department of Respiratory Medicine Ruijin Hospital Shanghai Jiaotong University School of Medicine Shanghai China; ^6^ Department of Respiratory Medicine Tongji Hospital Tongji Medical College Huazhong University of Science and Technology Wuhan China; ^7^ Department of Respiratory Medicine Second Xiangya Hospital Central South University Changsha China; ^8^ Department of Allergy and Clinical Immunology Guangzhou Institute of Respiratory Health The First Hospital Guangzhou Medical University Guangzhou China; ^9^ Department of Respiratory Medicine Zhongshan Hospital Fudan University Shanghai China; ^10^ Department of Pulmonary Medicine Second Affiliated Hospital of Wenzhou Medical University Wenzhou China; ^11^ Department of Pulmonary Medicine General Hospital of Northern Theater Command of the Chinese People's Liberation Army Shenyang China; ^12^ Department of Pulmonary Medicine Affiliated Sir Run Run Shaw Hospital Zhejiang University School of Medicine Hangzhou China; ^13^ Department of Pulmonary Medicine Tongji Hospital Tongji University School of Medicine Shanghai China; ^14^ Department of Pulmonary Medicine Qilu Hospital of Shandong University Jinan China; ^15^ Department of Pulmonary Medicine Henan Provincial People's Hospital People's Hospital of Zhengzhou University Zhengzhou China; ^16^ Department of Pulmonary Medicine Second Affiliated Hospital of Fujian Medical University Fujian China; ^17^ Department of Respiratory Disease Xijing Hospital The Fourth Military Medical University Xian China

**Keywords:** chest tightness variant asthma, inhaled corticosteroids, long‐acting beta‐agonists, real‐world study

## Abstract

**Background:**

Asthmatic patients with chest tightness as their only presenting symptom (chest tightness variant asthma [CTVA]) have clinical characteristics of eosinophilic airway inflammation similar to those of classic asthma (CA); however, whether CTVA has similar response to antiasthma treatment as compared with CA remains unclear.

**Objective:**

The response of 76 CTVA patients to standard asthma treatments with inhaled corticosteroids with long‐acting beta‐agonists was explored in a 52‐week multicenter, prospective, real‐world study.

**Results:**

After 52 weeks of treatment with therapy regimens used for CA, the mean 5‐point Asthma Control Questionnaire (ACQ‐5) score decreased markedly from 1.38(first administration) to 0.71 (52 weeks, mean decrease: 0.674, 95%CI: 0.447‐0.900, *P*<.001).The mean asthma quality‐of‐life questionnaire (AQLQ) score increased from 5.77 (first administration) to 6.20 (52 weeks, mean increase: 0.441, 95% CI 0.258‐0.625, *P*<.001). Furthermore, at week 52, FVC, FEV_1_%, the diurnal variation in PEFand the PD20‐FEV_1_ were significantly improved. Subgroup analysis revealed that the patients at first administration in the responsive group had higher ACQ‐5 scores than those in the nonresponsive group (*P* < .05).

**Conclusion:**

In conclusion, patients with CTVA had a good therapeutic response to the guideline‐recommended routine treatment (containing inhaled corticosteroids). The association between the treatment response and the severity of CTVA suggested that CTVA patients with higher ACQ‐5 scores had better therapeutic effects.

Dear editor,

In 2013, we have reported chest tightness being the only respiratory symptom among 24 asthmatic patients on presentation,^1^ and referred to this type of asthma as chest tightness variant asthma (CTVA). Compared with patients with classic asthma (CA) or cough variant asthma (CVA),^2^, ^3^ patients with CTVA also presented with eosinophilic airway inflammation. However, whether CTVA has similar response to antiasthma treatment as compared with CA remains unclear. We therefore sought to explore the therapeutic response to standard asthma treatments among 76 patients with CTVA in a 52‐week multicenter, prospective, real‐world study.

The study was conducted in 16 centers (see Supporting Information) in mainland China. Participants were recruited between April 1, 2015 and March 31, 2018 (Figure 1). We recruited treatment‐naive patients (14‐80 years of age) who had a history of chest tightness for at least 6 months. The definition of CTVA was made based on the chest tightness being the sole symptom and at least one of the following conditions was met: (a) an increase of >12% and >200 mL in forced expiratory volume in 1 s (FEV_1_) after inhaling salbutamol; (b) airway hyperresponsiveness as evidenced by a positive finding of bronchial provocation test; (c) a weekly variability in diurnal peak expiratory flow (PEF) of greater than 10%; and (d) a marked clinical improvement in response to β2 receptor agonists, with or without inhaled corticosteroids (ICS). All patients were treated with ICS plus long‐acting β2 receptor agonist based on the *Global Initiative for Asthma* (GINA) guidelines.

**FIGURE 1:**
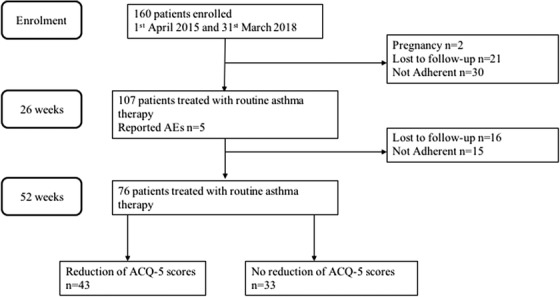
Flow chart showing the course of study ACQ‐5, 5‐item of Asthma Control Questionnaire.

The following baseline characteristics were collected from eligible patients: age, gender, the history of atopy, smoking status, body mass index (BMI), FEV_1_ (percent predicted), the ratio of FEV_1_/forced vital capacity (FVC), diurnal variation in PEF, the fraction of exhaled nitric oxide (FeNO), patient‐rated anxiety scale (SAS) and depression scale (SDS), Asthma Quality‐of‐Life Questionnaire (AQLQ), and the revised 5‐point Asthma Control Questionnaire (ACQ‐5)^4^ (see Supporting Information). The scores of the ACQ‐5 ranged from 0 to 6, with higher scores indicating poor asthma control (minimal clinically meaningful difference: 0.5).^5^, ^6^ Data were collected and recorded in a standardized file at the first administration, and at weeks 4, 13, 26, and 52.

The primary efficacy endpoint was the alterations in ACQ‐5 score after 52 weeks of treatment. Secondary efficacy endpoints were the alterations in FEV_1_, the provocative dose resulting in a 20% decrease in the FEV_1_ (PD20‐FEV_1_), diurnal variation in PEF, AQLQ, and the number of asthma attacks. Comparisons of ACQ‐5, AQLQ, SAS, FeNO, and lung function results were performed by using repeated‐measure one‐way analysis of variance. All statistical analyses were performed by Graph Prism software version 8.0.

Seventy‐six patients with CTVA were included (Table 1). At 52 weeks, chest tightness were significantly ameliorated among most patients with CTVA. The mean ACQ‐5 score (Figure 2A) decreased from 1.38 (first administration) to 0.71 (52 weeks) (mean decrease: 0.674; 95% confidence interval [CI], 0.447‐0.900; *P *< .001). The mean AQLQ score (Figure 2B) increased from 5.77 (first administration) to 6.20 (52 weeks) (mean increase: 0.441; 95% CI, 0.258‐0.625; *P *< .001). Only a single patient with CTVA had an asthma attack with cough during the treatment phase. Additionally, anxiety was also ameliorated after treatment (Figure 2C). Furthermore, at week 52, FVC, FEV_1_%, the diurnal variation in PEF, (Figure 2D‐F), and the PD_20_FEV_1_ were significantly improved (Table 2). However, there were no significant improvements in FeNO and FEV_1_ after 52 weeks compared with the baseline level.

**TABLE 1:** Demographic and clinical features of included subjects

Age (years)	
Mean	41.8 ± 12.1
Range	18‐68
Age group, no. (%)	
18‐30 years	14 (18.9)
31‐50 years	41 (55.4)
>50 years	19 (25.7)
Sex male, no. (%)	
Female	45 (59.2)
Male	31 (40.8)
BMI (kg/m^2^)	22.3 ± 2.8
Smoking status, no. (%)	
Current smoker	8 (11.8)
Former smoker	7 (10.3)
Never smoked	53 (77.9)
History of atopy, no. (%)	20 (27.4)
FEV_1_ % predicted	88.3 ± 16.4
FEV_1_/FVC %	78.5 ± 9.9
Blood eosinophils counts (× 10^9^ per L)	0.19 ± 0.23
FeNO (ppb)	26.2 ± 21.6
Anxiety and depression	
SAS score	2.1 ± 0.5
SDS score	2.1 ± 0.5
AQLQ	5.8 ± 0.8
ACQ‐5 score	1.4 ± 0.9

*Note*. Data are presented as mean ± SD or n (%). The ACQ‐5 assesses asthma symptoms in the previous weeks, each of which is scored on a 7‐point scale that ranges from 0 (no impairment) to 6 (maximum impairment) and averaged; a 0.5‐unit change represents the minimal clinically important difference.

Abbreviations: BMI, body mass index; FEV_1_, forced expiratory volume in 1 s; FVC, forced vital capacity; FeNO, fractional exhaled nitric oxide; SAS:, self‐rating anxiety scale; SDS, self‐rating depression scale; AQLQ, Asthma Quality‐of‐Life Questionnaire; ACQ‐5, the 5‐point Asthma Control Questionnaire.

**FIGURE 2:**
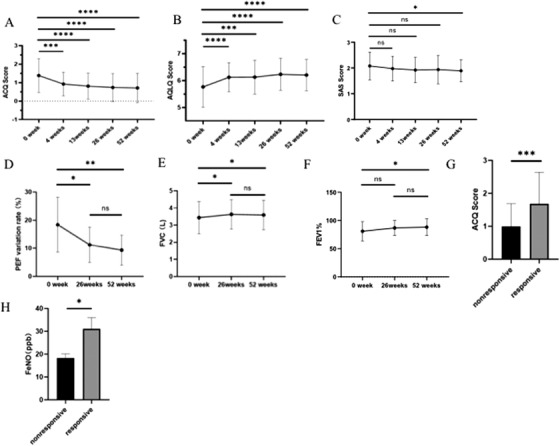
A–B, Time course of improvements in 5‐point Asthma Control Questionnaire (ACQ‐5) and Asthma Quality‐of‐Life Questionnaire (AQLQ) over a 52‐week period of treatments in chest tightness variant asthma (CTVA) patients. C, Changes in SAS scores over a 52‐week period of treatments in CTVA patients. D‐F, Changes in PEF variant rate (D), FVC (E), and FEV_1_% (F) over a 52‐week period of treatments in CTVA patients. G and H, The difference of ACQ (G) and FeNO (H) at week 0 between responsive and nonresponsive group. ^*^ *P* < .05; ^**^ *P* < .01; ^***^ *P* < .01; ^****^ *P* < .001

**TABLE 2:** The bronchial provocation test for CTVA patients

	52 weeks	
First administration	Negative (n)	Positive (n)
Negative (n)	8	1
Positive (n)	22	10

*Note*. Data are presented as n. The results of airway hyperreactivity for CTVA patients were all significantly improved after 1‐year treatment (*P* < .001). In the first administration, bronchial provocation test results showed that 32 patients were positive and nine were negative. After 52 weeks of treatment, 22 out of the 32 patients with an initial positive test finding achieved conversion.

Next, patients were divided into the responsive (43/76, 56%) and nonresponsive subgroups (33/76, 44%) according to the changes in ACQ‐5 score at 52 weeks (cutoff: 0.5). The responsive subgroup had higher ACQ‐5 scores and FeNO than those in the nonresponsive subgroup at the first administration (*P *< .05) (Figures 2G and 2H), suggesting that the therapeutic response was associated with the severity of CTVA.

Currently, various methods have been proposed to classify asthma control, including the ACQ‐5 score, GINA, or *Gaining Optimal Asthma Control* study criteria. However, no classification has been universally accepted. O'Byrne et al^7^ showed that, in a clinical trial setting, changes in the absolute ACQ‐5 score were significantly greater than those in the categorical scale. We have employed the revised ACQ‐5 to assess the asthma control status in patients with CTVA. The number of patients in the responsive group and nonresponsive group did not differ substantially (43 vs 33 cases). Nevertheless, the optimal treatment regimens for CTVA need to be further investigated in randomized controlled trials.

FeNO could be an airway eosinophilic biomarker for the assessment and management of asthma.^8^ In our study, FeNO at 52 weeks did not decrease significantly compared with that of the first administration. However, subgroup analysis revealed that FeNO at the first administration in the responsive group was markedly higher than that in the nonresponsive group.

Notably, anxiety was common in patients with CTVA, with the SAS score at 52 weeks being significantly lower than that at the first administration of therapy without concurrent treatments for anxiety or depression. Similarly, Kayaba et al demonstrated that patients with CVA were more depressed and anxious than the outpatients with CA.^9^

It has been demonstrated that cough, shortness of breath, or chest discomfort such as chest pain or tightness could be the isolated symptom of asthma.^10^, ^11^ Our findings reaffirmed that patients with asthma can present with a variety of symptoms. We did not set up CA and CVA control groups when exploring the therapeutic effect of CTVA, which should be regarded as the main limitation of our study.

In conclusion, patients with CTVA had a good therapeutic response to the guideline‐recommended routine treatment (containing ICS). The association between the treatment response and the severity of CTVA suggested that patients with CTVA who had higher ACQ‐5 scores would respond better to therapeutic interventions.

## ETHICS APPROVAL AND CONSENT TO PARTICIPATE

The study was approved by the Institutional Review Board for Human Studies of Second Affiliated Hospital of Zhejiang University School of Medicine, Hangzhou, China. ClinicalTrials.gov identifier: NCT 03237221.

## CONFLICT OF INTEREST

The authors declare no conflict of interest.

## Supporting information

SUPPORTING INFORMATION
