# Supplementary material for: Response of patients with chest tightness variant asthma with routine asthma treatment regimen: A 1‐year multicenter, prospective, real‐world study
Source: Clin Transl Med. 2020 Sep 15;10(5):e178. doi: 10.1002/ctm2.178 (PMC7503098; doi:10.1002/ctm2.178)
Supplement: Supplementary file 1 — SUPPORTING INFORMATION [file CTM2-10-e178-s001.docx]

1. The revised 5-point Asthma Control Questionnaire (ACQ-5)

Circle the number of the response that best describes how you have been during the past week.

1. On average, during the past week, how often were you woken by your chest tightness during the night?

0 Never

1 Hardly ever

2 A few minutes

3 Several times

4 Many times

5 A great many times

6 Unable to sleep because of chest tightness

2. On average, during the past week, how bad were your chest tightness symptoms when you woke up in the morning?

0 No symptoms

1 Very mild symptoms

2 Mild symptoms

3 Moderate symptoms

4 Quite severe symptoms

5 Severe symptoms

6 Very severe symptoms

3. In general, during the past week, how limited were you in your activities because of your chest tightness?

0 Not limited at all

1 Very slightly limited

2 Slightly limited

3 Moderately limited

4 Very limited

5 Extremely limited

6 Totally limited

4. In general, during the past week, how much of the time did you have chest tightness?

0 Not at all

1 Hardly any of the time

2 A little of the time

3 A moderate amount of the time

4 A lot of the time

5 Most of the time

6 All the time

5. On average, during the past week, how many puffs of short-acting bronchodilator (eg. Ventolin) have you used each day?

0 None

1 1±2 puffs most days

2 3±4 puffs most days

3 5±8 puffs most days

4 9±12 puffs most days

5 13±16 puffs most days

6 More than 16 puffs most days

2 16 centers involved in this study

1 Key Laboratory of Respiratory Disease of Zhejiang Province, Department of Respiratory and Critical Care Medicine, Second Affiliated Hospital of Zhejiang University School of Medicine, Hangzhou China,

2 Department of Respiratory Medicine, State Key Laboratory of Respiratory Disease, National Clinical Research Center for Respiratory Disease, Guangzhou Institute for Respiratory Health, The First Affiliated Hospital of Guangzhou Medical University,

3 Department of Pulmonary and Critical Care Medicine, affiliated Hospital, Institute of Respiratory Diseases, Guangdong Medical Univesity, Zhanjiang, China,

4 Department of Respiratory and Critical Care Medicine, Shenzhen People’s Hospital, Shenzhen, China,

5 Department of Respiratory and Critical Care Medicine, Ruijin Hospital, Shanghai Jiaotong University School of Medicine, Shanghai, China,

6 Department of pulmonary and critical medicine, Tongji Hospital, Tongji Medical College,  Huazhong University of Science and Technology, Wuhan, China,

7 Department of pulmonary and critical medicine, the second Xiangya Hospital, Central South University, Changsha, China,

8 Department of allergy and clinical immunology, Guangzhou Institute of Respiratory Health, The First Hospital, Guangzhou Medical University, Guangzhou, China,

9 Department of Pulmonary and Critical Care Medicine, Zhongshan Hospital, Fudan 10 University, Shanghai, 200030, China,

11 Department of Pulmonary Medicine, The Second Affiliated Hospital and Yuying Children's Hospital of Wenzhou Medical University, Wenzhou, Zhejiang, China,

12 Department of Pulmonary Medicine, Gneral Hospital of Northern Theater Command of the Chinese People’s Liberation Army, ShenYang, China,

13 Department of Pulmonary Medicine, Affiliated Sir Run Run Shaw Hospital, Zhejiang University School of Medicine, Hangzhou, China,

14 Department of Pulmonary and Critical Care Medicine, Tongji Hospital, Tongji University School of Medicine, Shanghai, China,

15 Department of Pulmonary and Critical Care Medicine, Qilu hospital of Shandong University,

16 Department of Pulmonary and Critical Care Medicine, Henan Provincial People's Hospital, People's Hospital of Zhengzhou University, Zhengzhou, China,

17 Department of Pulmonary and Critical Care Medicine, The Second Affiliated Hospital of Fujian Medical University, Fujian, China,

18 Department of Respiratory disease, Xijing Hospital, The Fourth Military Medical University.
